# Evaluating and optimizing ecological networks in the Hubei Yangtze river economic belt using an importance–sensitivity and circuit–graph approach

**DOI:** 10.1038/s41598-025-27304-2

**Published:** 2025-12-05

**Authors:** Junfeng Zeng, Shushen Li

**Affiliations:** College of Horticulture and Gardening, Yangtze University, Jingzhou, China

**Keywords:** Ecological network, The hubei section of the yangtze river economic belt, Ecosystem service importance, Ecological sensitivity, Circuit theory, Ecology, Ecology, Environmental sciences, Environmental social sciences

## Abstract

This study focuses on the Yangtze River Economic Belt in Hubei Province, addressing ecosystem service degradation and ecological risks under rapid urbanization. An integrated “Importance–Sensitivity–Circuit Theory–Graph Theory” framework was developed to optimize the regional ecological network. Ecological sources were identified by combining ecosystem service importance and ecological sensitivity. Circuit theory was applied to simulate multi-path corridors and key nodes, while graph theory quantitatively evaluated network connectivity. Results indicate that ecological sources show a spatial pattern of “high in the east and west, low in the middle,” with the Jianghan Plain identified as a key corridor break zone; Circuit theory revealed multiple potential corridors, high-priority pinch points, and barriers needing restoration; Graph analysis confirmed that the optimized network achieved significant improvements in closure, connection efficiency, and overall connectivity. By coupling ecological functional attributes with structural network optimization, this framework overcomes the limitations of traditional approaches that focus primarily on landscape morphology or single indicators, and its systematic and quantitative evaluation pathway provides a transferable methodological reference for constructing ecological networks in complex regions. The findings offer scientific support for regional ecological security pattern development and territorial spatial planning.

**Supplementary Information:**

The online version contains supplementary material available at 10.1038/s41598-025-27304-2.

## Introduction

Against the backdrop of global climate change and intensifying human activities, regional landscape patterns are undergoing dramatic reorganization, making habitat fragmentation and ecosystem degradation major threats to biodiversity and ecological security^1–3^. Rapid economic and social development has sharpened the contradiction between resource exploitation and ecological carrying capacity^4,5^, placing ecosystems under increasing pressure and amplifying their vulnerability and instability^6,7^. In this context, scientifically identifying and conserving key ecological spaces to construct structurally integral and functionally stable ecological networks is strategically crucial for safeguarding ecological security, harmonizing human-land relationships, and promoting regional sustainable development^8^.

Ecological network construction follows a well-established technical paradigm centered on the framework of “ecological source identification – resistance surface construction – corridor extraction” ^9,10^. In ecological source identification, early studies primarily relied on Morphological Spatial Pattern Analysis (MSPA), selecting natural reserves and forest patches as the main ecological sources^11–13^. With the advancement of ecological research, the focus has progressively shifted from morphological characteristics to functional attributes. Subsequent approaches have incorporated indicators such as habitat distribution^14,15^, ecosystem service value^16,17^, ecosystem service importance(ESI)^18–20^, ecological sensitivity(ES)^21–23^, and landscape connectivity^24,25^, either individually or in combination. This methodological transition has driven the evolution of source identification from a morphology-based assessment toward one emphasizing ecological functionality and process integrity. Resistance surface construction has progressed from single-factor proxies (e.g., land use type)^26^ to multi-factor integrations that combine natural conditions (e.g., topography, vegetation) and anthropogenic disturbances (e.g., road density, night-time light) to improve their accuracy^27,28^. For corridor extraction, the Minimum Cumulative Resistance (MCR) model^8,12,13^ has been increasingly supplemented by Circuit Theory, which simulates random-walk processes to identify multiple pathways, pinch points, and barriers, thus more realistically capturing species movement complexity^18,29^. Furthermore, graph theory analysis and connectivity indices (e.g., α, β, γ) are now widely adopted to quantify network stability, identify key nodes, and prioritize restoration, enhancing both the efficacy and operability of ecological security patterns^30,31^.

However, persistent limitations in current research hinder the accuracy and efficacy of ecological networks. In ecological source identification, the transition from single-factor to multi-dimensional approaches has not yet resolved a fundamental issue: the lack of systematic coupling between ecosystem service supply (importance) and system vulnerability (sensitivity). This disconnect often leads to a spatial delineation of sources that is misaligned with the integrated demands of complex ecological processes. During ecological network construction, the widespread application of circuit theory is often undermined by its reliance on MSPA-derived morphological patches as source inputs. Even when ecosystem service or sensitivity indicators are incorporated, they are frequently treated as auxiliary corrective factors rather than being systematically embedded into the functional processes that circuit theory aims to simulate. Consequently, the resulting networks overemphasize structural connectivity at the expense of a more ecologically meaningful functional connectivity. Furthermore, quantitative assessments of network structure—including the identification of critical nodes and vulnerable links—remain underdeveloped. There is a notable scarcity of studies that provide a robust quantitative comparison of network performance before and after optimization.

To address these gaps, this study introduces an integrated analytical framework that couples ESI and ES in a dual-dimensional manner for source identification. This approach prevents the overestimation of low-sensitivity, high-service areas and the oversight of high-sensitivity, high-risk zones, thereby significantly improving delineation accuracy. Building on this foundation, we jointly employ circuit theory and graph theory to establish a systematic pathway from source identification and corridor simulation to network optimization and evaluation. Through this methodological integration, the framework forms a closed-loop process of “precise identification → realistic simulation → quantitative evaluation,” offering a more reliable scientific foundation for constructing ecological networks in complex human-environment systems.

The Hubei section of the Yangtze River Economic Belt (HYREB), situated in central China, serves as the core section of the Yangtze River Economic Belt and functions as a crucial ecological barrier and economic hub connecting the upper and lower reaches. However, with the rapid advancement of urbanization and industrialization, ecological land in this region has been increasingly encroached upon, leading to intensified fragmentation of farmland, forestland, and wetlands, continuous degradation of ecosystem service functions, and heightened ecological risks. In particular, the Jianghan Plain in the central part of the region, characterized by intensive agricultural production and a fragile ecological background, faces severe corridor disruptions, which have become a critical bottleneck for regional ecological connectivity^20,32^. Consequently, systematic research on constructing and optimizing ecological networks across the entire HYREB remains scarce.

Based on the above background and theoretical framework, this study takes the HYREB as the study area. By comprehensively considering ESI, ES, and landscape connectivity, it constructs a regional ecological network and proposes optimization strategies. The research objectives are as follows: (1) to identify ecological sources that possess both high ecological value and key spatial node functions; (2) to reveal the spatial pathways and barrier patterns of ecological flows; and (3) to quantitatively evaluate network connectivity and optimization effectiveness. The results of this study can provide scientific support for the construction of regional ecological security patterns, the implementation of ecological restoration, and territorial spatial planning.

## Study area and data

### Study area

**Fig. 1:**
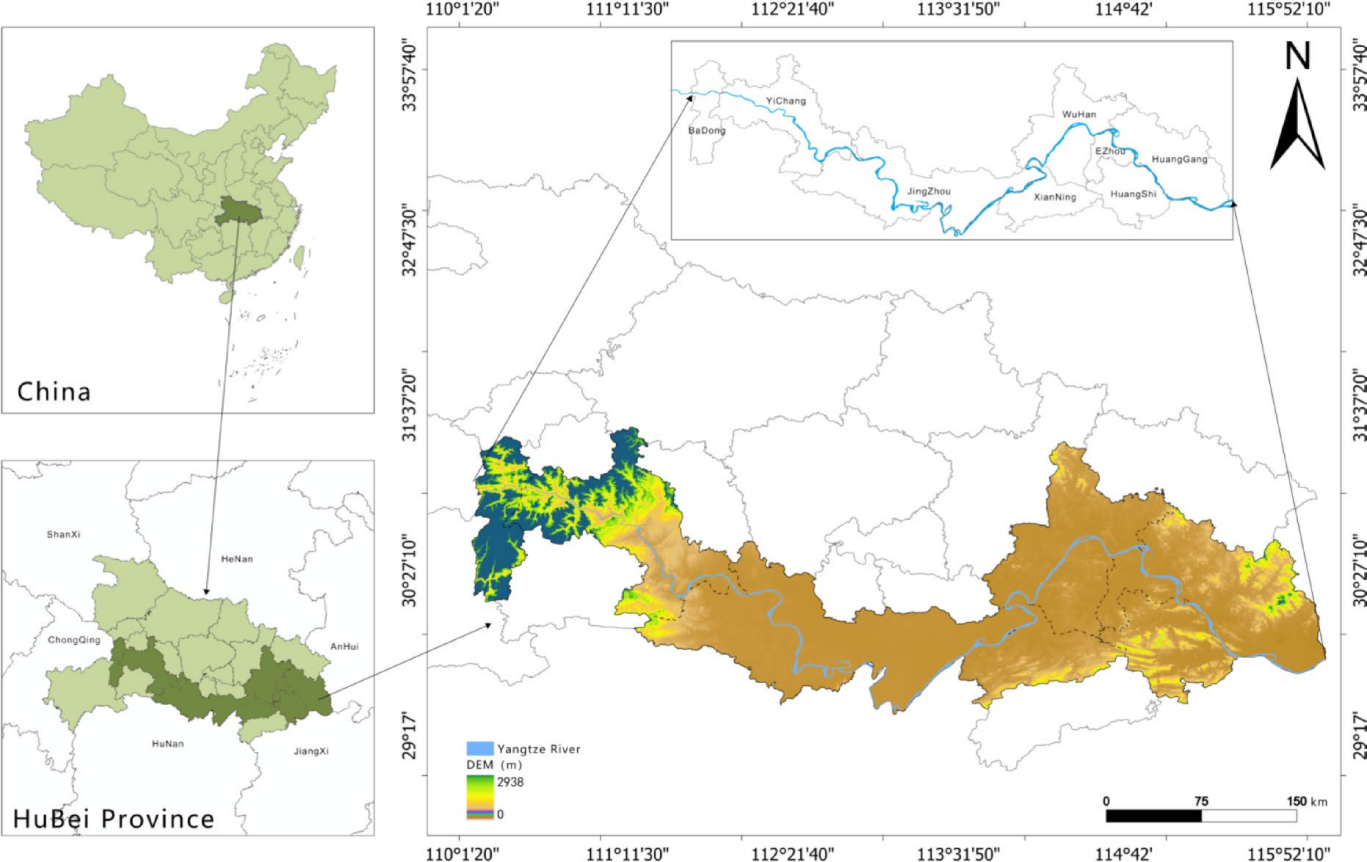
The study area. It was drawn by ArcGIS 10.7 (https://desktop.arcgis.com/zh-cn/desktop/index.html).

The HYREB is situated in central China (110°23′–118°28′ E, 26°29′–31°51′ N). Encompassing eight prefecture-level cities along the Yangtze River’s main stream, it covers a total area of approximately 54,160 km² (Fig. 1). The region’s geomorphology is characterized by a distinct “high in the east and west, low in the center” pattern. The western area is dominated by the forested Wuling and Daba mountain ranges. The central Jianghan Plain features an extensive network of rivers and lakes, while the eastern part transitions into the southern foothills of the Dabie Mountains and hilly wetlands. Hosting eight national and four provincial nature reserves, the HYREB constitutes a vital ecological security barrier for the middle-lower Yangtze River basin and plays a strategic role in maintaining regional ecological balance and fostering coordinated socio-economic development.

### Data sources and processing

The sources of various data involved in this article are shown in Table 1, and All data were uniformly coordinated to the WGS1984_UTM_Zone_50N geographic coordinate system, and all data were resampled to 30 m×30 m to facilitate data clipping and calculation.

**Table 1:** Data source.

Data name	Data type	Resolution(m)	Time range	Data source
LULC	Raster	30	2020	http://www.geodata.cn
DEM	Raster	30	2020	http://www.gscloud.cn
Precipitation	Raster	1000	2020	http://www.geodata.cn
Population density	Raster	1000	2020	https://www.resdc.cn
NDVI	Raster	1000	2020	https://www.resdc.cn
Evaporation amount(ET)	Raster	500	2020	http://www.geodata.cn
Harmonized world soils database v2.0	Raster	1000	2023	https://gaez.fao.org/
Roads	Vector	-	2020	https://www.resdc.cn

## Methods

This study developed an integrated framework for constructing and optimizing the ecological network. The methodology comprised five key steps: (1) assessing ESI by integrating the InVEST model and the Analytic Hierarchy Process (AHP); (2) evaluating ES based on eight selected indicators; (3) identifying ecological sources by overlaying evaluation results and selecting patches with high dPC values; (4) constructing an ecological resistance surface and simulating corridors using Circuit Theory; and (5) analyzing network topology via graph theory and proposing spatial optimization strategies.

### Evaluation of ESI

**Fig. 2:**
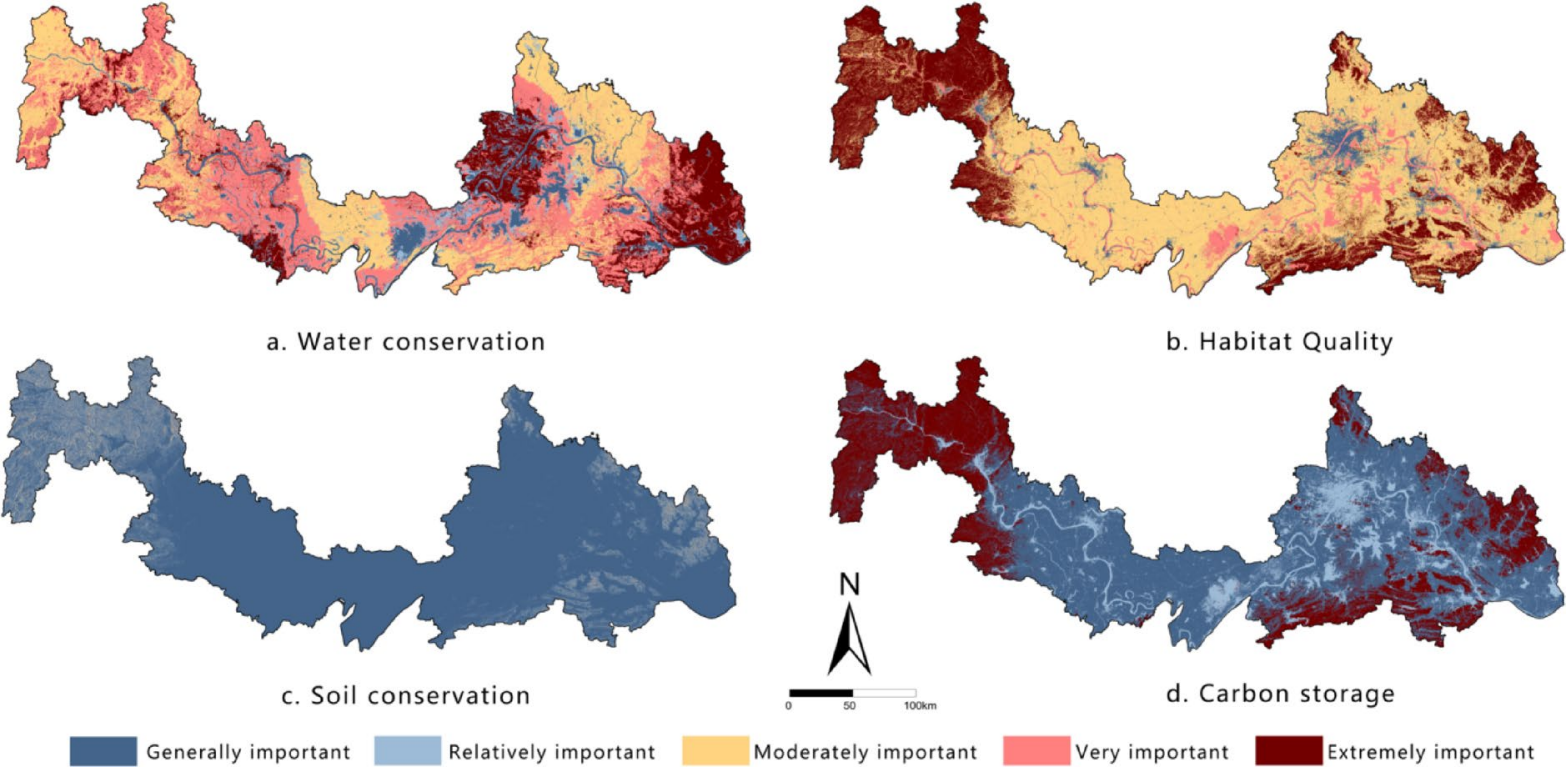
Spatial distribution of importance levels for each factor. It was drawn by ArcGIS 10.7 (https://desktop.arcgis.com/zh-cn/desktop/index.html).

ESI refers to the critical and irreplaceable role of ecosystems in maintaining and delivering service functions^33^.Given the abundant rainfall, dense network of rivers and lakes, and rich water resources in the HYREB—along with high vegetation coverage and well-preserved biodiversity in the eastern and western mountainous regions—this study selected four key ecosystem services as evaluation indicators: water conservation, habitat quality, soil conservation, and carbon storage. The InVEST model was used to quantify the spatial distribution and functional intensity of each service^34^. and the Natural Breaks (Jenks) method was applied to classify results into five levels (Fig. 2).

To quantify the relative importance of various ecosystem services, this study employed the Analytic Hierarchy Process (AHP) to determine their relative weights^35^. Nine domain experts were invited to evaluate the relative importance of each indicator using a 9-point scale (see Supplementary Table S1). All constructed judgment matrices (see Supplementary Table S2) passed the consistency check (CR < 0.1, see Table S4), ensuring the rationality and reliability of the weight assignments. The calculated weights (Table 2) indicated that water conservation (29.76%) and soil retention (27.48%) were assigned the highest weights, highlighting their central role in maintaining regional ecological security. Finally, a weighted overlay analysis was performed for all ecosystem service indicators on the ArcGIS platform to generate a synthesized ESI map. The results were classified into five categories to visually represent the spatial distribution characteristics of ESI.

**Table 2:** Weights of each importance factor.

Factors	Weights
Water conservation	29.76%
Habitat quality	23.55%
Soil conservation	27.48%
Carbon storage	19.21%

### ES assessment

**Fig. 3:**
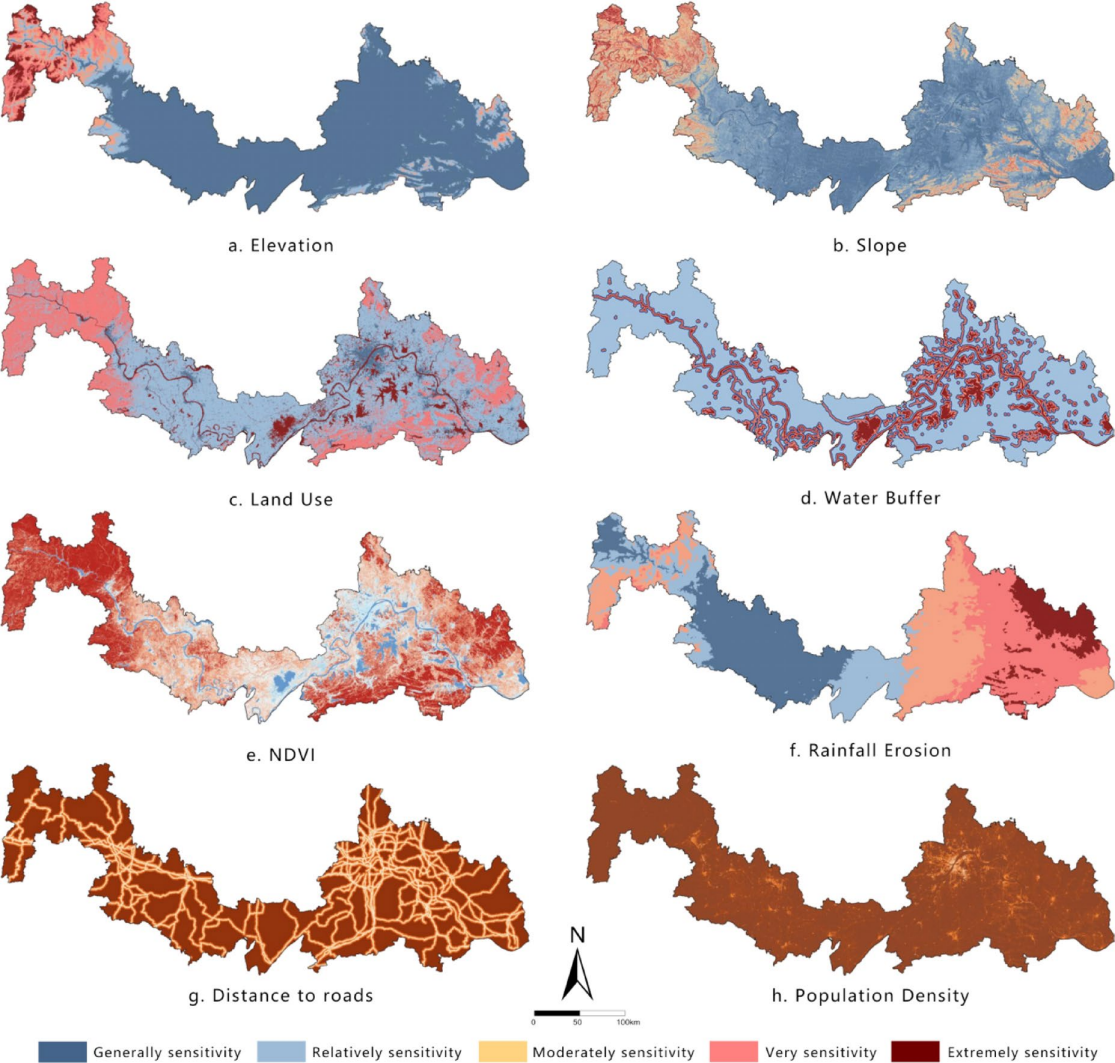
Spatial distribution of sensitivity levels for each factor. It was drawn by ArcGIS 10.7 (https://desktop.arcgis.com/zh-cn/desktop/index.html).

ES reflects the degree to which ecosystems respond to natural environmental changes and human disturbances, revealing the likelihood of ecological risks and the vulnerability characteristics of a region^36^. Based on the characteristics of the study area, eight indicators were selected to construct the ecological sensitivity evaluation system: elevation, slope, land use, vegetation cover (NDVI), rainfall erosivity, Water buffer, distance to roads, and population density. Each factor was classified using the Natural Breaks method (Fig. 3) to capture spatial heterogeneity.

The weighting method for ES was the same as that for ESI, both using the Analytic Hierarchy Process (AHP). All judgment matrices (see Supplementary Tables S3) passed the consistency test (CR < 0.1, see Table S4), ensuring the rationality and reliability of the assigned weights. The weight results (Table 3) show that vegetation cover (27.93%) and land use type (15.69%) had the greatest influence on ecological sensitivity, followed by rainfall erosivity (13.97%) and population density (13.43%), indicating that both natural surface characteristics and human disturbances are dominant drivers of ecological sensitivity. A comprehensive ecological sensitivity map was produced using weighted summation in ArcGIS, and classified into five sensitivity levels with the Natural Breaks method.

**Table 3:** Weights of each sensitivity factor.

Factors	Weights
Elevation	5.44%
Slope	6.41%
Water buffer	12.33%
Land use	15.69%
NDVI	27.93%
Rainfall erosion	13.97%
Distance to roads	4.80%
Population density	13.43%

### Evaluation of landscape connectivity

Landscape connectivity provides a quantitative measure of the degree of linkage among important patches, reflecting the spatial structure of ecological elements within a landscape. Two core indices^37^, *dPC* (patch importance index) and *dIIC* (delta Integral Index of Connectivity), are commonly used to quantify landscape connectivity and the contribution of individual patches to maintaining overall connectivity^24,25^.

(1) The patch importance index (*dPC*), derived from the Probability of Connectivity (*PC*), measures the decrease in overall landscape connectivity resulting from the removal of a specific patch. It evaluates the critical role of a patch in facilitating species movement or ecological flows^38^. The index is calculated as follows:

1 $$\:\text{P}\text{C}=\frac{{\sum\:}_{\text{i}=1}^{\text{n}}{\sum\:}_{\text{j}=1}^{\text{n}}{\text{a}}_{\text{i}}{\text{a}}_{\text{j}}{\text{P}}_{\text{i}\text{j}}^{\ast\:}}{{\text{A}}_{\text{L}}^{2}}\:\:\:\:\:\:\:\:\:\:\:\:\:\:$$

where *n* represents the total number of patches in the study area, $\:{a}_{i}$ and $\:{a}_{j}$ are the areas of patches *i* and *j*, $\:{P}_{ij}^{\ast\:}$ is the maximum connectivity probability between patches *i* and *j*, and $\:{A}_{L}^{}$ is the total landscape area. Higher *PC* values indicate better overall connectivity.

2 $$\:\text{d}\text{P}\text{C}=\frac{\text{P}\text{C}-{\text{P}\text{C}}_{\text{r}\text{e}\text{m}\text{o}\text{v}\text{e}}}{\text{P}\text{C}}\:\:\times\:100\text{\%}\:\:\:\:\:\:\:\:\:\:\:\:\:\:\:$$

Where $\:{PC}_{remove}$ is the *PC* value after removing patch. The *dPC* value indicates the relative importance of the patch in maintaining landscape connectivity; a larger *dPC* implies higher significance.

(2) The delta Integral Index of Connectivity (*dIIC*), based on the Integral Index of Connectivity (*IIC*), represents the proportion of connectivity loss in the topological network after the removal of a patch. It highlights the structural role of a patch as a connectivity hub^38^. The calculation is as follows:

3 $$\:\text{I}\text{I}\text{C}=\frac{\sum\:_{\text{i}=1}^{\text{n}}\sum\:_{\text{j}=1}^{\text{n}}\frac{{\text{a}}_{\text{i}}\cdot\:{\text{a}}_{\text{j}}}{1+\text{n}{\text{l}}_{\text{i}\text{j}}}}{{\text{A}}_{\text{L}}^{2}}$$

4 $$\:\text{d}\text{I}\text{I}\text{C}=\frac{\text{I}\text{I}\text{C}-\text{I}\text{I}{\text{C}}_{\text{r}\text{e}\text{m}\text{o}\text{v}\text{e}}}{\text{I}\text{I}\text{C}}\times\:100\text{\%}$$

where n is the total number of patches, $\:{a}_{i}$ and $\:{a}_{j}$ are patch areas, $\:{A}_{L}^{}$​is the total landscape area, and $\:{IIC}_{remove}$ is the IIC after removing patch. *dIIC* values greater than 1% are generally considered indicative of critical patches.

### Ecological source identification

To scientifically identify ecological sources that combine high ecological value with critical spatial nodes, this study integrated the evaluation results of ESI and ES. Areas classified as moderate, high, and extremely important/sensitive were extracted, and the top 30 patches by area were selected as initial candidate sources. Landscape connectivity analysis was then conducted using Conefor 2.6 software^38^. On the basis of previously published approaches^39–41^, the optimal distance threshold for our analysis was determined by comparing landscape connectivity indices (IIC and PC) at varying thresholds from 500 m to 10,000 m. This value was established at 5000 m, which corresponds to a dispersal probability of 0.5. Our result is consistent with those reported in other studies performed on a comparable spatial scale^42,43^. Finally, patches with a dPC value greater than 1% were ultimately designated as ecological sources. In total, 12 ESI sources and 15 ES sources were identified, together forming the spatial foundation of ecological sources in the study area.

### Ecological resistance surface construction

**Fig. 4:**
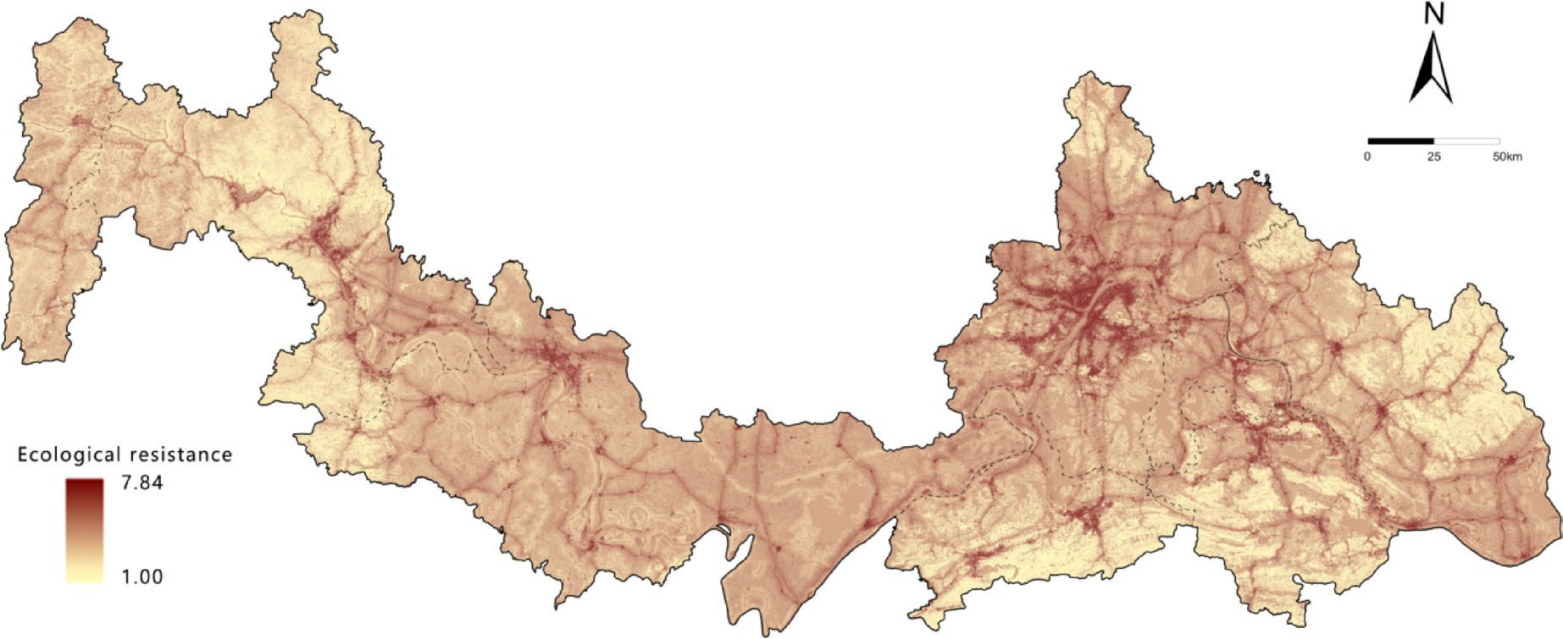
Spatial distribution of ecological resistance surfaces. It was drawn by ArcGIS 10.7 (https://desktop.arcgis.com/zh-cn/desktop/index.html).

The ecological resistance surface represents the landscape impediments species encounter during migration^44^, and its accuracy is crucial for reliable ecological network construction. Based on regional characteristics, seven resistance factors were selected: four natural environment factors (elevation, slope, distance to water bodies, and vegetation cover) and two human activity factors (land use type and distance to roads). Each factor was reclassified into five resistance levels using the 1–9 scale method (1, 3, 5, 7, 9) ^45^. The resistance weights were determined through an expert questionnaire survey, and the results were normalized in SPSS to obtain the relative weights of each factor (Table 4). The weighted resistance factors were overlaid in ArcGIS to produce the final ecological resistance surface (Fig. 4), which served as the spatial foundation for corridor extraction.

**Table 4:** Resistance factor classification criteria and weight Assignment.

Resistance factors	Resistance value					Weights
	1	3	5	7	9	
elevation	< 500	500–1000	1000–1500	1500–2000	> 2000	0.14
slope	< 10	10–20	20–40	40–60	> 60	0.10
land use type	Forest, Wetland	Grassland, Water	Cropland, Shrub	Barren	Impervious	0.25
vegetation cover	> 0.7	0.5–0.7	0.3–0.5	0.1–0.3	< 0.1	0.30
distance to water bodies	> 1500	800–1500	300–800	100–300	< 100	0.10
distance to roads	> 2000	1000–2000	500–1000	100–500	< 100	0.11

### Ecological network construction based on circuit theory

Circuit theory, by simulating the random-walk behavior of electrons, effectively models species migration processes in heterogeneous landscapes^18^. This approach not only identifies core ecological corridors but also quantifies their spatial extent and connectivity strength, offering multi-dimensional support for ecological network planning. In this study, the Linkage Mapper toolbox in ArcGIS 10.7 was utilized to extract ecological corridors and identify key nodes based on predefined ecological sources and resistance surfaces^22^. The specific procedure included: (1) determining potential ecological corridors using the Build Network and Map Linkages tool to calculate least-cost paths; (2) identifying key ecological pinch points with high current density using the Pinchpoint Mapper tool in all-to-one mode; and (3) detecting ecological barrier points using the Barrier Mapper tool in Maximum mode, with a moving window search radius set to 1000 m based on repeated trials.

### Ecological network evaluation

Graph theory was employed to abstract the landscape into a network model for quantitative assessment. Three topological indices—network closure (α), node-to-link ratio (β ), and connectivity (γ) ^30,31^—were used to evaluate the overall structure of the ecological network in the HYREB. The formulas are as follows:

5 $$\:{\upalpha\:}=\frac{\text{L}-\text{V}+1}{2\text{V}-5}$$

6 $$\:{\upbeta\:}=\frac{\text{L}}{\text{V}}$$

7 $$\:{\upgamma\:}=\frac{\text{L}}{3\left(\text{V}-2\right)}$$

Where L represents the number of corridors and V the number of nodes. The α - index ranges from 0 to 1, with higher values indicating stronger circuit connectivity; the β - index ranges from 0 to 3, reflecting network complexity; and the γ - index ranges from 0 to 1, where higher values suggest more efficient node connectivity.

## Results and analysis

### Spatial distribution characteristics of ESI and ES

Evaluation results (Table 5) show significant differences in the classification of ESI and ES. The ESI classification exhibits a spindle-shaped pattern: the “Relatively important” class dominates (43.45%), forming the ecological matrix of the region; the “Very” and “Moderately important” classes serve as important supplements (totaling approximately 35%); while the “Extremely important” class, representing core ecosystem services, accounts for the smallest proportion (only 7.95%), highlighting the limited spatial extent of high-value core areas. In contrast, the ES classification demonstrates high vulnerability: the intermediate classes (“Relatively,” “Moderately,” and “Very” sensitive) are evenly distributed (totaling over 70%), and the “Extremely sensitive” class is prominent (18.53%), far exceeding the proportion of the “Extremely important” ESI class. Meanwhile, the lowest sensitivity class (“Generally sensitive”) accounts for the smallest proportion (10.30%), further confirming the overall vulnerability of the regional ecosystem. The results indicate that the ecological functions of the study area are primarily of medium importance, but ES is generally high, particularly with the spatial coverage of highly vulnerable areas (“Extremely sensitive”) significantly larger than that of core functional zones (“Extremely important”). This contradictory pattern underscores that regional development must strictly avoid highly sensitive and vulnerable areas, prioritize ecological protection measures, and use their carrying capacity thresholds as the core basis for spatial regulation.

**Table 5:** Area statistics of ESI levels and ES levels.

ESI			ES		
Level	Area/km2	Proportion	Level	Area/km2	Proportion
Generally	7326.4	13.53%	Generally	5576.45	10.30%
Relatively	23,529	43.45%	Relatively	13873.38	25.62%
Moderately	8189.28	15.12%	Moderately	13921.06	25.71%
Very	10807.2	19.96%	Very	10751.78	19.85%
Extremely	4304.55	7.95%	Extremely	10033.74	18.53%

The “Relatively important” class of ESI accounts for 43.45%, forming the regional ecological matrix, while the “Extremely sensitive” class of ES accounts for 18.53%—far higher than the 7.95% of “Extremely important” ESI. This indicates that the study area’s ecosystem is highly vulnerable, consistent with Su et al.’s (2021) findings on the Jianghan Plain’s ecological sensitivity^20^.

**Fig. 5:**
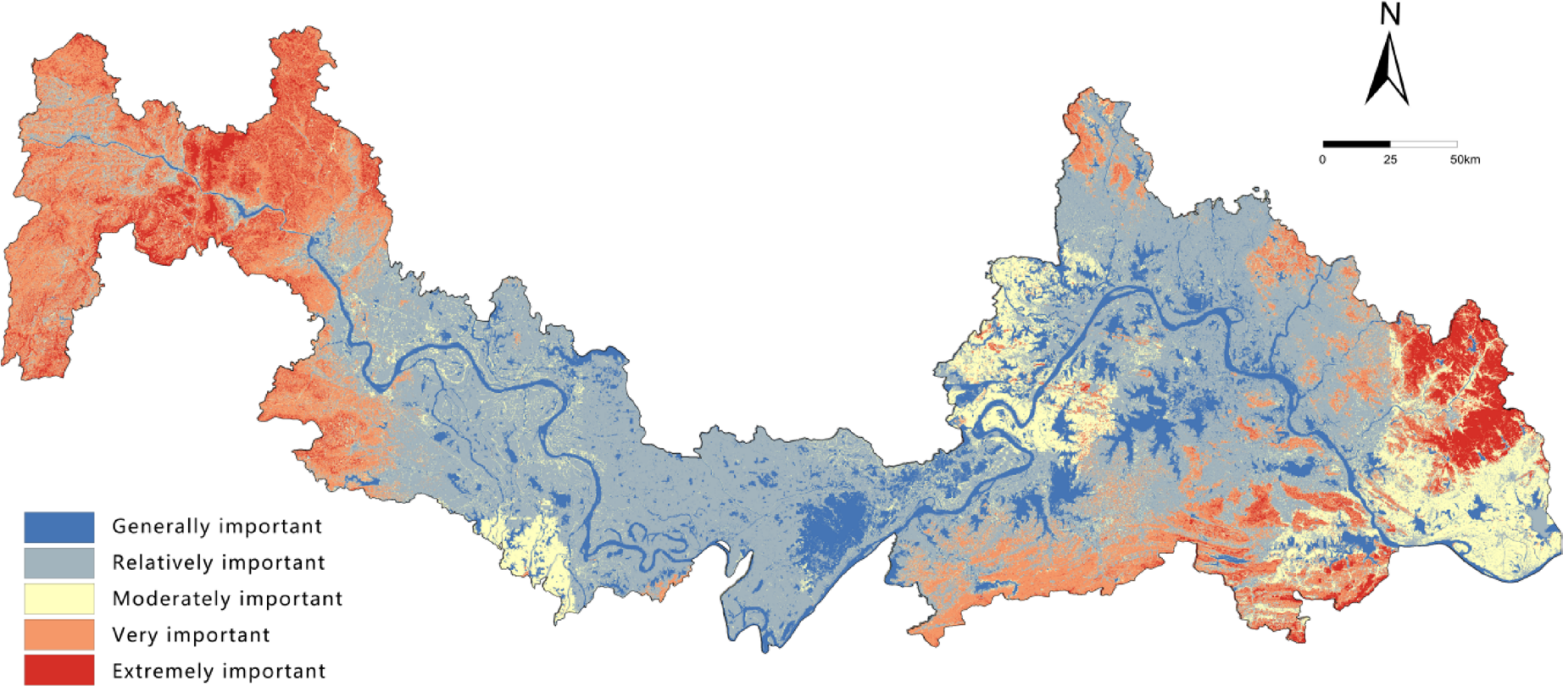
Spatial distribution of ESI. It was drawn by ArcGIS 10.7 (https://desktop.arcgis.com/zh-cn/desktop/index.html).

**Fig. 6:**
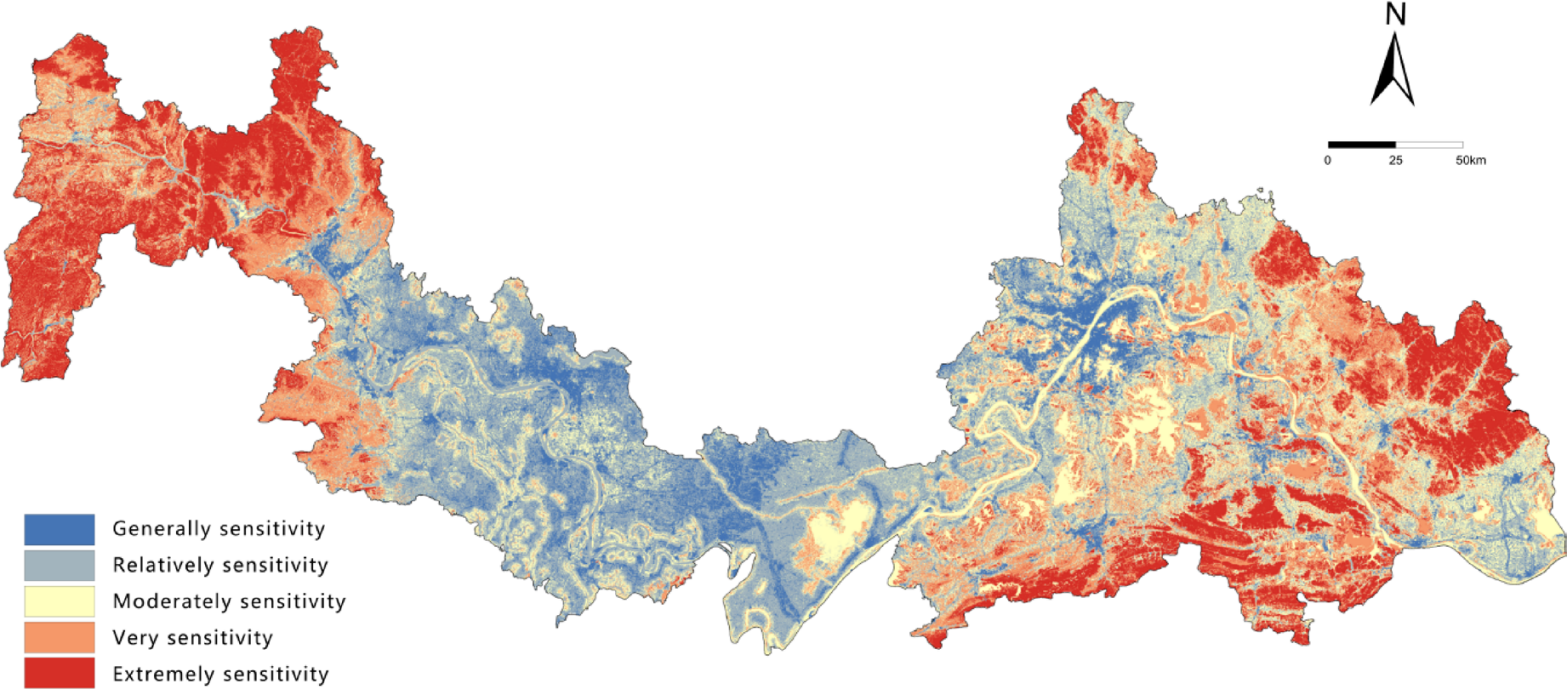
Spatial distribution of ES. It was drawn by ArcGIS 10.7 (https://desktop.arcgis.com/zh-cn/desktop/index.html).

Spatially (Figs. 5 and 6), both ESI and ES exhibit a macro-scale pattern of “high in the east and west, low in the center.” The central Jianghan Plain, dominated by cropland, forms a distinct low-value belt, while the mountainous areas in the east and west serve as high-value core zones. Notably, the “Extremely important” and “Extremely sensitive” areas show significant spatial overlap, forming clustered blocks in three typical regions: the western area (Enshi-Badong County to western Yichang City, in the Wuling and Qinba Mountains), the eastern area (eastern Huanggang, Huangshi, and southern Xianning, extending from the Dabie Mountains), and local ecological nodes in northern Wuhan. The land cover types in these high-value areas are highly consistent, dominated by forest ecosystems in the Qinba, Wuling, and Dabie Mountains, supplemented by natural wetlands, indicating high naturalness. The results reveal that although the cropland-dominated Jianghan Plain has lower ecological ratings, the natural ecological network connecting the eastern and western mountains through mountain ranges forms an ecological barrier system traversing the watershed. This pattern highlights a synergistic division of labor: “mountainous areas dominate ecological supply, while plains undertake agricultural production,” providing important insights for coordinating conservation and development in the regional.

ESI shows a distinct “high in the east and west, low in the middle” pattern. High-value areas are mainly distributed in the Dabie Mountains in the east and the Wuling–Qinba Mountains in the west, while the Jianghan Plain in the central region forms a continuous low-value belt dominated by farmland.

ES exhibits a similar “high in the east and west, low in the middle” pattern. The eastern Dabie Mountains and western Wuling–Qinba Mountains maintain high sensitivity due to relatively intact ecosystems, while the central Jianghan Plain shows lower sensitivity overall but still contains moderately sensitive areas within agricultural landscapes.

### Identification of ecological sources

Based on the previous analyses and to enhance the scientific validity and effectiveness of ecological network construction, this study incorporated landscape connectivity indices (dPC and dIIC) and the natural landscape pattern of the study area to identify 12 ESI sources and 15 ES sources. The total area of these sources is 12,147.62 km², accounting for 22.43% of the study area. Among them, ESI sources cover 8,549.24 km², and ES sources cover 6,700.69 km². Their overlapping areas form important–sensitive composite sources, covering 3,102.31 km² and representing 25.54% of the total ecological source area, highlighting their comprehensive and critical role in the regional ecological security pattern.

In terms of characteristics, some ESI sources are outstanding in both area and connectivity (Table 6), such as sources No. 10, 4, and 2, which are core patches maintaining regional ecosystem service functions. Particularly, source No. 10 is the largest (1,671.62 km²), with dPC and dIIC values reaching 33.46% and 33.67%, respectively, playing a significant role in supporting the overall connectivity of the ecological network. Some smaller sources (e.g., No. 11 and 5), although limited in their spatial role, serve as important supplements for maintaining local connectivity and enhancing the completeness of the overall network.

**Table 6:** Area and connectivity indices of ES source Patches.

No.	Area/km²	dPC/%	dIIC/%
1	587.26	4.12	4.16
2	1143.93	15.69	15.77
3	464.78	2.62	2.60
4	1296.29	19.96	20.25
5	358.45	1.67	1.55
6	459.13	2.70	2.54
7	543.47	3.57	3.56
8	532.67	3.46	3.42
9	581.43	4.70	4.07
10	1671.62	33.46	33.67
11	295.34	1.13	1.05
12	614.87	4.50	4.55

This table presents the area and connectivity importance (dPC and dIIC) of ESI source patches. Larger patches such as sources 10, 4, and 2 exhibit significantly higher connectivity values, serving as core nodes that sustain the overall ecological service network.

In contrast, ES sources have more distinct spatial distributions and functional attributes (Table 7). While providing certain ecosystem services, these sources are more susceptible to human activities and external disturbances, exhibiting higher ecological vulnerability and potential risks. For example, source No. 15 (907.34 km²) and No. 22 (669.21 km²) have dPC values exceeding 10%, playing notable roles in maintaining local ecological corridors. Damage to these sources could lead to local fragmentation or overall degradation of the ecological network, thereby amplifying regional ecological risks.

**Table 7:** Area and connectivity indices of ESI source Patches.

No.	Area/km²	dPC/%	dIIC/%
13	537.82	6.96	6.87
14	252.47	1.98	1.51
15	907.34	19.76	19.55
16	644.28	9.75	9.85
17	204.88	1.20	1.00
18	386.55	3.55	3.70
19	511.54	6.21	6.27
20	198.81	1.00	0.94
21	638.64	9.80	9.69
22	669.21	10.72	10.64
23	417.43	4.05	4.14
24	553.40	7.07	7.27
25	202.82	1.37	0.98
26	328.60	2.50	2.57
27	246.90	1.42	1.45

This table summarizes the area and connectivity importance (dPC and dIIC) of ES source patches. Larger patches such as sources 15 and 16 show notably high connectivity values, indicating their key roles in maintaining the integrity of the ecological network.

**Fig. 7:**
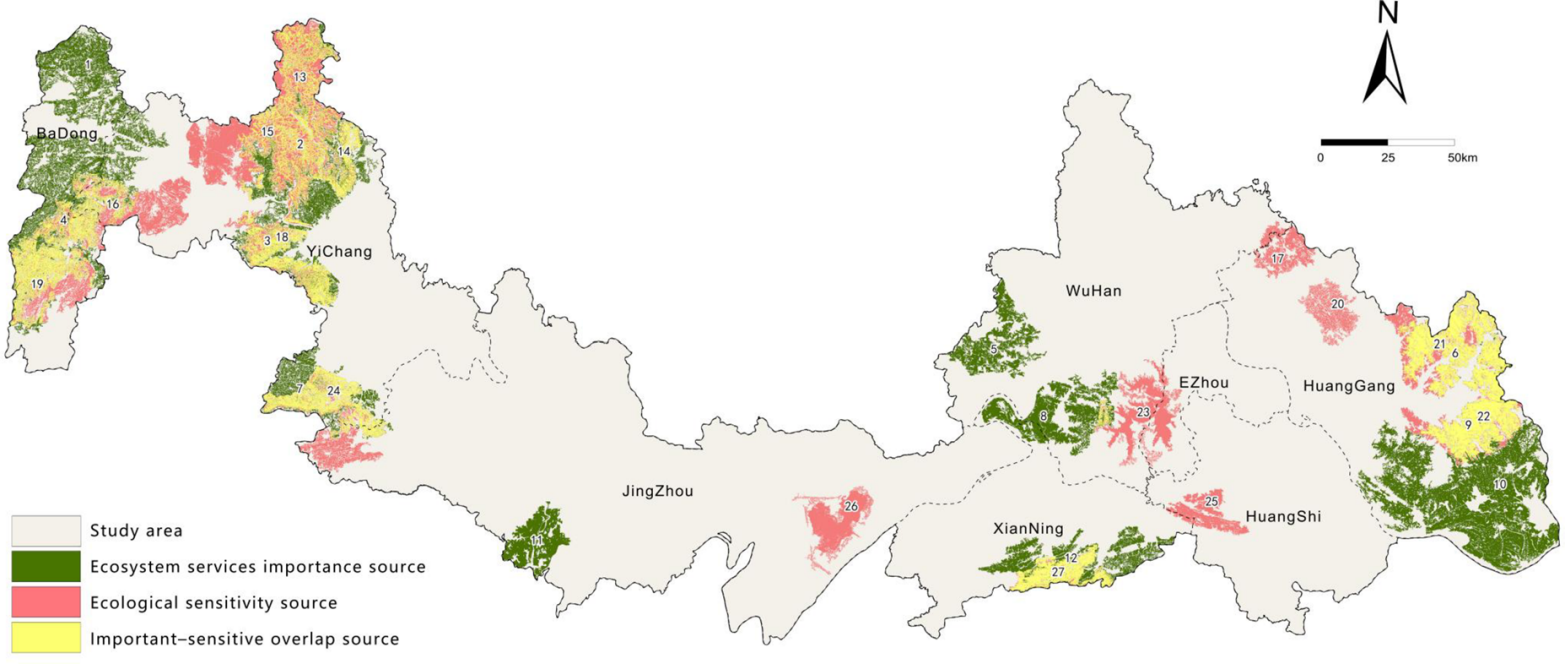
Spatial distribution pattern of ecological sources. It was drawn by ArcGIS 10.7 (https://desktop.arcgis.com/zh-cn/desktop/index.html).

Regarding the spatial distribution pattern of ecological sources (Fig. 7), ESI sources show significant spatial aggregation, mainly distributed in areas with well-preserved natural ecology, such as the western Wuling Mountains extension and Qingba Mountains, the southern Mufu Mountains, and the eastern Dabie Mountains. They are generally concentrated and highly connected, forming the main backbone of the ecological network. In contrast, the central area has smaller, fewer, and weakly connected ecological sources, representing potential weak points in the network that require restoration and reinforcement through ecological corridor construction. ES sources are more scattered and clustered in ecologically sensitive, development-pressured, or frequently disturbed areas, such as the central Honghu Lake and Liangzi Lake basins and some hilly regions. Although some high-risk sources also exhibit strong connectivity, their vulnerability to disturbance and complexity should not be overlooked, and their protection management and risk early warning should be strengthened in subsequent ecological network optimization.

Overall, ESI sources are the core support areas of the regional ecological security pattern, while ES sources are potential risk areas requiring key prevention and control. The composite ecological sources formed by their overlap possess both high value and high risk and should be prioritized for protection and management in ecological network construction and optimization.

ESI sources are concentrated in well-conserved mountains (western Wuling and Qinba, southern Mufu, eastern Dabie), forming the ecological network backbone. ES sources are scattered in sensitive or human-disturbed areas, mainly in central Honghu and Liangzi Lake basins. Overlap sources combine high value and high vulnerability, and should be prioritized for protection.

In summary, the identification of ESI sources and ES sources, together with their connectivity analysis, not only clarifies the key nodes in ecological network construction but also provides a scientific basis for subsequent corridor layout and functional zoning.

Ecological Network Construction.

**Fig. 8:**
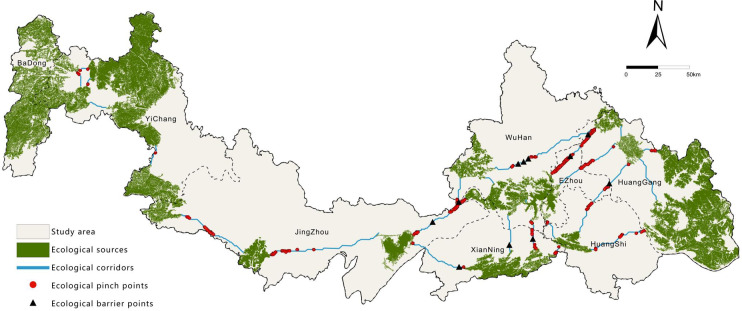
Ecological network of the Study Area. It was drawn by ArcGIS 10.7 (https://desktop.arcgis.com/zh-cn/desktop/index.html).

Based on ecological source and resistance surface data, a total of 22 core ecological corridors were identified using the Linkage Mapper tool (Fig. 8), with a combined length of 858.03 km. The average corridor length is 26.81 km, ranging from a minimum of 1 km to a maximum of 93.08 km. Spatially, these corridors demonstrate a pronounced “many at both ends, few in the middle; long in the east, short in the west” pattern. High corridor density is observed in the eastern region (encompassing Wuhan, Huanggang, Ezhou, Huangshi, and Xianning) and the western area (around Yichang), whereas the central region exhibits significant gaps. In the east, large and densely clustered ecological sources support a long-distance corridor network that functions as the backbone of regional ecological connectivity. In the west, closely grouped ecological sources give rise to short, high-density corridors with robust connectivity. The central-southern part contains only one long corridor (93.08 km), which traverses the agriculturally and urbanized Jianghan Plain—a region under high fragmentation pressure due to intensive human activities. The central-northern area lacks ecological sources entirely, resulting in a complete disruption of corridors that impedes species migration and ecological flows. This spatial pattern underscores the strong dependence of corridor distribution on the configuration of ecological sources. Ecological restoration and source reconstruction in the central region are urgently needed to enhance the integrity of the overall network.

Furthermore, based on circuit theory and the Barrier Mapper tool, 85 ecological pinch points (total area: 128.45 km²) and 11 ecological barriers (total area: 586.34 km²) were identified (Fig. 8). Pinch points are predominantly concentrated in the eastern part of the study area, with a scattered presence in the central and western regions. Their sizes vary between 0.64 km² and 12.23 km², and land cover is mainly composed of forest, wetland, water, and grassland—typically located in areas with relatively low resistance. These patches play a critical role in maintaining landscape connectivity, and their degradation would substantially increase the risk of ecological network fragmentation. Ecological barriers are largely clustered in the eastern cities of Wuhan, Xianning, and Huanggang, with none present in the west. The largest barrier spans 304.06 km² and consists primarily of water bodies, cropland, and artificial surfaces, highlighting the obstructive impact of areas with intensive human activity on ecological processes. Therefore, it is imperative to enhance the protection and restoration of ecological pinch points, and to mitigate or redirect the blocking effects of ecological barriers, thereby improving the integrity and functional stability of the regional ecological network.

22 ecological corridors were extracted, showing dense networks in eastern and western mountains but gaps in the central plain. Circuit theory and Barrier Mapper identified 85 pinch points (128.45 km²) crucial for connectivity and 11 barrier points (586.34 km²) obstructing ecological flows, highlighting key areas for protection and restoration.

### Analysis of ecological network structural quality

Based on graph theory, the structural quality of the ecological network in the study area is assessed as relatively weak (α = 0.15, β = 1.23, γ = 0.44), indicating poor circuitry, moderate connection efficiency, and low overall connectivity. The low α-index reflects a scarcity of closed loops within the network, where most corridors form linear branches. This results in limited alternative pathways for ecological flows and reduced system resilience. The moderate β-index suggests a relatively high number of connections between nodes, implying the potential emergence of a multi-core network structure centered around areas such as Honghu Lake and Liangzi Lake. Meanwhile, the low γ -index highlights inadequate overall connectivity and a relatively fragmented network, particularly between the central plains and the eastern and western mountainous regions. These structural weaknesses constrain species migration and genetic exchange.

As a result, the current ecological network is insufficient to effectively support regional ecological security. A strategy of “augmenting sources and reinforcing pathways” is recommended, focusing on restoring ecological sources and corridors within the Jianghan Plain, alongside enhanced protection of critical nodes to improve the functionality and stability of the network.

### Optimization of the ecological network in the study area

Based on the previous analysis, the central and northeastern parts of the study area lack ecological sources with high ESI or high ES. As a result, these regions fail to form effective ecological corridors that support species migration and energy flow, significantly compromising the integrity of the watershed ecological network and landscape connectivity.

To optimize the network structure, this study scientifically identified four key areas as supplementary ecological sources through a comprehensive evaluation of ecological importance and sensitivity, along with an analysis of the landscape connectivity index. These include the Changhu Wetland, Baling Mountain National Forest Park, and Mayu Mountain Forest Farm in the central region, as well as the Mulan Ecological Tourism Area in the northeast (see Table 8). The incorporation of these areas aims to address structural ecological gaps and enhance the overall functionality and connectivity of the ecological network.

**Table 8:** Supplementary source area Strategies.

No.	Name	Area/km²	Reason for selection
1	Baling Mountain National Forest Park	20.27	High ecological importance; moderate area; strong connectivity; key central hub lacking corridors; requires protection and source development.
2	Changhu Wetland	67.99	High ecological value and sensitivity; large area; strong landscape connectivity.
3	Mayu Mountain Forest Farm	16.76	High-value ecological services; development necessary.
4	Mulan Ecological Tourism Area	36.33	High ecological sensitivity; moderate area; strong landscape connectivity.

**Fig. 9:**
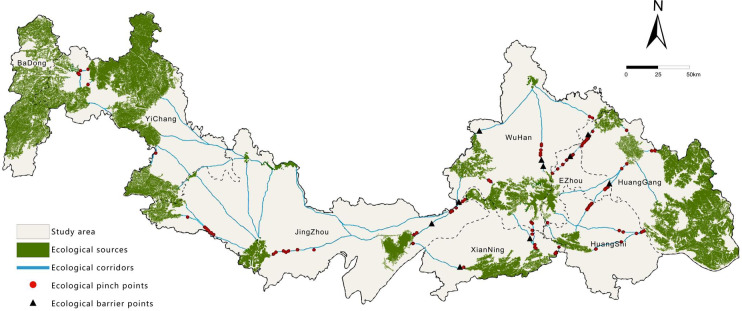
Optimized Ecological Network of the Study Area. It was drawn by ArcGIS 10.7 (https://desktop.arcgis.com/zh-cn/desktop/index.html).

By prioritizing ecological development in the supplemented source areas, their ecological functions and nodal efficiency can be effectively enhanced, allowing them to become key zones for species migration and ecological flow exchange in the central-northern part of the watershed. On this basis, the supplemented source areas were systematically integrated into the existing ecological network, and ecological corridors were re-extracted using the Linkage Mapper toolkit. This process ultimately formed an integrated ecological network system that connects east to west and provides full regional coverage (Fig. 9). The optimized network significantly enhances the structural stability and functional continuity of the watershed’s ecological security pattern.

After optimization, the number of core corridors increased to 37, notably adding multiple corridors in the central Jianghan Plain, enhancing connectivity and filling gaps in the previously fragmented region.

Structural analysis of the optimized ecological network based on graph theory showed increases in the α, β, and γ indices to 0.49, 1.87, and 0.67, respectively, with all three metrics demonstrating significant improvement compared to the pre-optimized state. Specifically, the 226% increase in the α-index indicates a substantial rise in circuit pathways and ecological loops, reflecting a qualitative leap in network resistance to disturbance and system recovery capacity. The 52% increase in the β-index suggests that the new ecological sources have effectively filled critical gaps in the original topology, resulting in richer node connectivity. The γ-index now exceeds the functional threshold of 0.6, marking the transition of the watershed ecological network from a state of “discrete patches” to an “integrated functional network”. Comprehensive analysis indicates that the optimized ecological network has achieved remarkable improvements in structural stability, spatial connectivity, and functional coordination, laying a solid spatial foundation for the sustained provision of ecosystem services.

## Discussion

### Identification of ecological sources and spatial pattern characteristics

This study identified ecological sources based on two dimensions: ESI and ES, overcoming the limitations of single-criterion evaluations and providing a more comprehensive reflection of the complexity of the regional ecosystem. The results show that ecological sources are concentrated in areas such as the Qinba Mountains, Wuling Mountains, Dabie Mountains, and Honghu Lake, whereas the central Jianghan Plain—due to intensive agricultural and urban development and fragmented ecological land—has become an area lacking ecological sources, forming a spatial pattern of “high in the east and west, low in the center.” This distribution is highly consistent with the spatial arrangement of natural ecological elements such as mountains, woodlands, and water bodies in the study area, confirming not only the core ecological function of mountainous regions but also revealing the structural ecological vulnerability of agricultural and urban zones. The formation of this spatial pattern is primarily driven by the combined effects of natural topographic gradients and the intensity of human activities. In the eastern and western mountainous areas, high forest coverage and low human disturbance contribute to stable ecosystem structures and well-maintained ecological functions. In contrast, the central Jianghan Plain is characterized by flat terrain, intensive agricultural practices, and the radiating influence of rapid urbanization from the Wuhan metropolitan area (including cities such as Xiantao, Tianmen, and Qianjiang). These factors have exacerbated the fragmentation of forest and wetland patches, significantly reducing ecological connectivity and making this region a weak link within the overall ecological network. The findings align with those of previous studies^18,21,46^, indicating that the two-dimensional identification method is more objective and reasonable compared to commonly used approaches for delineating ecological sources.

A noteworthy observation is that water bodies such as the Yangtze River and Han River received relatively low scores in the ESI evaluation but were mostly rated as “moderately sensitive” in the sensitivity assessment. This discrepancy reflects the essential difference between “service function” and “system vulnerability”: the service evaluation emphasizes terrestrial processes, leading to an underestimation of the service value of water bodies, whereas the sensitivity evaluation accurately captures their inherent susceptibility to human disturbance. This finding highlights the need to develop differentiated conservation strategies, implementing defensive measures focused on avoiding disturbance for highly sensitive water bodies.

### Structural characteristics and optimization effects of the ecological network

The ecological corridors extracted based on circuit theory exhibit a spatial pattern of “dense at both ends and sparse in the middle, longer in the east and shorter in the west,” reflecting the significant constraint imposed by the distribution of ecological sources on corridor formation. Connectivity is better in the concentrated source areas in the eastern and western regions, while the central plain—affected by both a lack of sources and intensive human activities—faces high risks of corridor fragmentation. Identified pinch points and barriers are mostly distributed within urban agglomerations (such as Wuhan, Jingzhou, Huangshi, and Ezhou), where ecological and anthropogenic conflicts are prominent, necessitating prioritized management and control.

By supplementing key ecological nodes in the central region, such as Changhu Wetland and Baling Mountain Forest Park, the connectivity indices of the network (α, β, and γ) were significantly improved. This demonstrates that the strategy of “augmenting sources and reinforcing pathways” can effectively optimize the structure of the ecological network. These small and medium-sized ecological patches serve as “stepping stones” within the region, playing a crucial role in mitigating ecological fragmentation^47^.

### Comparison with traditional methods

More accurate ecological source identification. Compared with traditional single-indicator approaches, the integrated framework proposed in this study—“dual-dimension identification (ESI + ES) – circuit theory – graph-theoretic analysis”—demonstrates clear advantages in source identification, corridor simulation, and network evaluation and optimization.

 Conventional methods for identifying ecological sources often rely on a single indicator, such as MSPA, ESI, or ES. This approach struggles to comprehensively capture the multifunctionality and intrinsic vulnerability of ecosystems, potentially leading to one-sided assessments and the oversight of ES or high-risk areas. This study overcomes these limitations by integrating two dimensions—ESI and ES—to identify sources from both “supply” and “risk” perspectives. This dual-dimension approach not only ensures the adequate spatial representation of crucial ecosystem services but also significantly enhances the identification accuracy of ecologically sensitive and potentially high-risk zones. Consequently, it makes the selection of ecological sources more rational and scientifically grounded for constructing regional ecological security patterns.

More realistic corridor simulation and ecological flow representation. The traditional Minimum Cumulative Resistance (MCR) model, which is based on the “single optimal path” assumption^12^, struggles to simulate the multi-path dispersal processes of ecological flows and fails to effectively identify potential bottlenecks and critical barriers in the landscape network. For example, in the ecological network constructed by Guo et al. (2025) for the Jianghan Plain using the MCR model^48^, although multiple linear corridors were identified, the study did not further pinpoint barrier segments prone to disruption or ecologically critical pinch point areas where ecological flows are highly concentrated. In contrast, the circuit theory applied in this study conceptualizes the landscape as a conductive network and uses current density to effectively simulate the spatial diffusion probability of ecological flows. The results not only identify the main pathways of ecological movement but also simultaneously reveal key ecological pinch points and barriers that significantly affect connectivity^18^. This comprehensive identification capability provides a more holistic and actionable scientific basis for the precise optimization of the ecological network and the prioritization of restoration efforts.

More systematic network quality assessment.While traditional evaluations of ecological networks often rely on qualitative descriptions of overall disturbance or node-specific metrics like dPC and BC^49,50^, they lack a holistic framework for quantifying overall network structure. To address this gap, this study employs graph theory indices (α, β, γ) to provide a quantitative and systematic assessment of the network’s connectivity, complexity, and integrity before and after optimization. The results demonstrate that the optimized network achieves enhanced overall resilience, a more balanced structure, and richer ecological flow paths, thereby offering a robust scientific foundation for constructing regional ecological security patterns.

In summary, the integrated “dual-dimension identification – circuit theory – graph-theoretic analysis” framework achieves a systematic integration of source identification, flow simulation, and network optimization on a theoretical level. More importantly, it demonstrates superior precision and operational feasibility in practical application, presenting a novel technical pathway for regional ecological spatial governance and corridor optimization.

### Uncertainties and future prospects

Despite the integrative nature of our framework, which combines ESI, ES, circuit theory, and graph-based analysis, the findings are subject to several uncertainties. These primarily stem from (1) the parameterization of the resistance surface, where expert-based assignments for land-use types may influence corridor delineation; (2) the scale dependency of spatial resolution, as finer data (e.g., 30 m) may capture noise while coarser data (e.g., 100 m) might obscure narrow corridors; and (3) potential inaccuracies and temporal mismatches in underlying environmental datasets, which could bias the assessment of ecosystem services and source identification. Furthermore, the validation of modeled corridors remains challenging due to limited field observations of ecological flows.

Future research should prioritize the empirical validation of corridor patterns against independently observed wildlife movement data or NDVI time-series to assess the ecological stability of identified sources. To enhance the model’s robustness and policy relevance, subsequent studies should also focus on refining resistance calibration with field-survey data and implementing multi-scenario simulations that incorporate projected land-use and climate changes.

### Policy recommendations

To systematically enhance regional ecological resilience, this study proposes a tailored strategy—“Reconnecting the East, Enhancing the Center, and Protecting the Flanks”—for the Hubei section of the HYREB, based on its “high in the east and west, low in the center” topographic and ecological gradient. This strategy aims to establish a sustainable ecological security pattern through the following regional measures:

In the Eastern and Western Mountainous Zones, the priority is strict protection to preserve ecosystem integrity and reinforce natural barriers. This will be achieved by enforcing stringent controls on human activities and implementing ecological conservation projects. These efforts are essential for ensuring long-term ecological stability and facilitating the synergistic development of eco-tourism and forest carbon sequestration.

In the Central Plains, the focus shifts to functional enhancement through Nature-based Solutions (NbS). Centering on major lakes such as Honghu and Changhu, key measures include converting low-yield farmland to wetlands, establishing ecological shelterbelts, and rehabilitating fragmented waterways. The objective is to create an integrated wetland-farmland ecosystem network that significantly improves the region’s capacities for hydrological regulation and biodiversity conservation.

In the Eastern Urban Agglomerations, where ecological barriers are most concentrated, the paramount goal is restoring connectivity. Targeted actions include implementing corridor reconnection projects, constructing greenways to mitigate barriers, and installing ecological overpasses across major roads. These interventions are designed to directly counteract habitat fragmentation and restore ecological flows disrupted by intensive human development.

The coordinated implementation of these zone-specific strategies will systematically build a sustainable ecological security pattern. This integrated approach is fundamental to bolstering long-term ecological resilience and safeguarding the continuous provision of vital ecosystem services.

## Conclusion

This study systematically constructed and optimized the ecological network of the HYREB by integrating ESI evaluation, ES analysis, circuit theory, and graph theory. The main conclusions are as follows:

1. A total of 12 ESI source areas and 15 ES source areas were identified, covering a total area of 12,147.62 km² (accounting for 22.43% of the study area). Among these, the overlapping areas of high service importance and high sensitivity accounted for 3,102.31 km² (25.54% of the total source area). 22 core ecological corridors were extracted, with a total length of 858.03 km and an average length of 26.81 km. The spatial distribution of corridors exhibited a pattern of “longer in the east, shorter in the west.”
2. The ecological network demonstrated a structure characterized by “high ecological quality in the eastern and western regions and low connectivity in the central region.” The mountainous areas of Qinba, Wuling, and Dabie formed the backbone of the network, while the central Jianghan Plain—dominated by agricultural land—exhibited low ecological connectivity, representing a critical ecological barrier.
3. Targeted restoration of ecological sources and corridors in the central plain significantly improved network connectivity. Graph theory indices increased from pre-optimization (α = 0.15, β = 1.23, γ = 0.44) to post-optimization (α = 0.49, β = 1.87, γ = 0.67), indicating notable enhancements in network circuitry, complexity, and overall connectivity.

The integrated framework of “ecosystem service importance–ecological sensitivity–circuit theory–graph theory” proposed in this study is applicable for systematically constructing and optimizing ecological networks in urban–agricultural mosaic landscapes. Future research should strengthen corridor validation based on species-specific functional activities and incorporate multi-scenario simulations to enhance the practicality and adaptability of ecological network planning.

## Supplementary Information

Below is the link to the electronic supplementary material.

Supplementary Material 1

## Data Availability

The datasets used and analysed during the current study available from the corresponding author on reasonable request.
