# Supplementary material for: Evaluating and optimizing ecological networks in the Hubei Yangtze river economic belt using an importance–sensitivity and circuit–graph approach
Source: Sci Rep. 2025 Dec 5;15:43220. doi: 10.1038/s41598-025-27304-2 (PMC12680631; doi:10.1038/s41598-025-27304-2)
Supplement: Supplementary file 1 — Supplementary Material 1 [file 41598_2025_27304_MOESM1_ESM.docx]

Table S1. Details of the Expert Panel

| **Expert ID** | **Affiliation** | **Title** | **Research Area** |
| --- | --- | --- | --- |
| **E1** | Yangtze University | PhD, Professor | Landscape Ecology |
| **E2** | Yangtze University | PhD, Professor | Urban Planning |
| **E3** | Yangtze University | PhD, Professor | Landscape Architecture |
| **E4** | Yangtze University | PhD, Associate Professor | Landscape Ecology |
| **E5** | Yangtze University | PhD, Associate Professor | Landscape Architecture |
| **E6** | Yangtze University | PhD, Associate Professor | Yangtze River ecological research |
| **E7** | Huazhong Agricultural University | PhD, Professor | Landscape Ecology |
| **E8** | Huazhong Agricultural University | PhD, Professor | Landscape Architecture |
| **E9** | Beijing Urban Planning & Design Institute | PhD, Professor | Landscape Ecology |

*To protect the privacy of the experts, only the Expert ID, affiliation, title, and research area are listed; personal names are not provided.

Table S2. AHP Matrix of Ecosystem Services Importance

| **Factors** | **Water conservation** | **Habitat quality** | **Soil conservation** | **Carbon storage** |
| --- | --- | --- | --- | --- |
| **Water conservation** | 1 | 1.261 | 1.144 | 1.468 |
| **Habitat quality** | 0.793 | 1 | 0.833 | 1.259 |
| **Soil conservation** | 0.847 | 1.201 | 1 | 1.468 |
| **Carbon storage** | 0.681 | 0.794 | 0.681 | 1 |

Table S3. AHP Matrix of Ecological Sensitivity

| **Factors** | **Elevation** | **Slope** | **Water buffer** | **Land use** | **NDVI** | **Rainfall erosion** | **Distance to roads** | **Population density** |
| --- | --- | --- | --- | --- | --- | --- | --- | --- |
| **Elevation** | 1 | 0.760 | 0.360 | 0.457 | 0.206 | 0.310 | 1.136 | 0.470 |
| **Slope** | 1.316 | 1 | 0.467 | 0.451 | 0.224 | 0.338 | 1.639 | 0.480 |
| **Water buffer** | 2.780 | 2.143 | 1 | 0.494 | 0.414 | 1.201 | 2.647 | 0.776 |
| **Land use** | 2.188 | 2.218 | 2.024 | 1 | 0.610 | 1.015 | 3.113 | 1.096 |
| **NDVI** | 4.852 | 4.460 | 2.415 | 1.639 | 1 | 2.536 | 5.629 | 1.955 |
| **Rainfall erosion** | 3.225 | 2.962 | 0.833 | 0.986 | 0.394 | 1 | 2.450 | 1.050 |
| **Distance to roads** | 0.880 | 0.610 | 0.378 | 0.321 | 0.178 | 0.408 | 1 | 0.375 |
| **Population density** | 2.126 | 2.082 | 1.289 | 0.913 | 0.511 | 0.952 | 2.664 | 1 |

Table S4. Summary of Consistency Test Results

| **Evaluation System** | **Maximum Eigenvalue** | **CI Value** | **RI Value** | **CR Value** | **Consistency Check Result** |
| --- | --- | --- | --- | --- | --- |
| **Ecosystem Services Importance** | 4.002 | 0.001 | 0.890 | 0.001 | Pass |
| **Ecological Sensitivity** | 8.104 | 0.015 | 1.410 | 0.011 | Pass |
